# Neural model for multi-stability in visual action recognition

**DOI:** 10.1186/1471-2202-16-S1-P279

**Published:** 2015-12-18

**Authors:** MA Giese, L Fedorov, R Vogels

**Affiliations:** 1 Department of Cognitive Neurology, Section Computational Sensomotorics, CIN & HIH, University Clinic Tübingen, Germany; 2 Laboratorium voor Neuro-en Psychofysiologie, KU Leuven, Leuven, Belgium

## Introduction:

The visual perception of body movements shows interesting dynamical properties. Biological motion perception can show perceptual multi-stability with respect to the perceived walking direction [1]. In addition, body motion perception is subject to adaptation, as is demonstrated by the existence of high-level after-effects and of fMRI repetition suppression in relevant areas. Existing neural models for action recognition do not account for these phenomena. We present neurodynamical model that reproduces these phenomena, and which allows to study the interplay between multi-stability, adaptation and intrinsic fluctuations in body motion perception.

## Methods:

The core of the model is a two-dimensional neural field [2], whose first dimension encodes the time-course of the action stimulus (in terms of snapshot sequences) while the second dimension encodes the stimulus view. In addition, inspired by results on adaptation processes in area IT [3], the model includes two different types of adaptation processes, modeling firing rate fatigue and input fatigue, and a Gaussian noise process. The model is fitted to psychophysical data on multi-stability in action perception, and the details of the adaptation processes were fitted, exploiting electrophysiological results from area IT. The multi-stability of the 2D neural field model can be mathematically analyzed based on a novel approach that is based on level-sets [4].

## Results / Discussion:

A version of the model for the representation of static stimuli reproduces quantitatively electrophysiological results on neural adaptation in area IT [3]. The model accounts in a unifying way for multiple neurodynamic phenomena in action recognition: (i) temporal sequence-selectivity for movies with different sequential orders of the stimulus frames; (ii) perceptual multi-stability for silhouette stimuli that are compatible with multiple views; (iii) sizes of the electrophysiologically observed adaptation effects for the repeated presentation of the same action stimulus [5,6]. A detailed quantitative analysis shows that perceptual switches in the neural dynamics are primarily induced by fluctuations (noise), not by neural adaptation. In addition, the model predicts a new highly efficient stimulus for the demonstration of adaptation effects in action perception, which induces stronger adaptation within the neural representation than the previously applied stimuli. Finally, it turns out that adaptation effects induced by action stimuli are critically dependent on the neural mechanism of adaptation. Firing-rate fatigue mechanisms seem to result in much smaller adaptation effects for dynamic stimuli than mechanisms based on input fatigue, when the adaptation effects for static stimuli are matched.
